# Mental well-being in residents of different regions of Russia

**DOI:** 10.1192/j.eurpsy.2021.1085

**Published:** 2021-08-13

**Authors:** R. Shilko, L. Shaigerova, O. Almazova, A. Dolgikh

**Affiliations:** Faculty Of Psychology, Lomonosov Moscow State University, Moscow, Russian Federation

**Keywords:** sociocultural mediation, regions of Russia, mental health, mental well-being

## Abstract

**Introduction:**

Some attempts are being made to describe the mental health and well-being of the population in relation with the regional specifics in a particular area of the Russia, however, they are rare and local (limited to one region).

**Objectives:**

The current study aims to identify sociocultural mediation of mental well-being based on its measures in Russia’s regions with different ethno-cultural composition of population.

**Methods:**

The study involved 210 men and 403 women aged 14 to 76 years (M = 26.9; SD = 13.7) from six regions of the Russian Federation: Moscow, St. Petersburg, Udmurtia, Sakha, Sverdlovsk and Kemerovo. The mental well-being of participants was assessed using the Warwick-Edinburgh Mental Wellbeing Scale (Tennant et al., 2006; Tennant et al., 2007).

**Results:**

The measures of mental well-being were different in the regions: Moscow (M=51.04; SD=8.03), St.-Petersburg (M=50.05; SD=5.78), Udmurtia (M=47.57; SD=9.50), Sakha (M=50.99; SD=8.47), Sverdlovsk (M=54.86; SD=8.43), Kemerovo (M=51.84; SD=7.51). Using one-way analysis of variance (ANOVA), it was found that there are significant differences in the assessment of psychological well-being between study participants from different regions (F = 6.692; p <0.001). Residents from the Sverdlovsk evaluate their mental well-being as significantly better than the ones from Moscow (MD=3.821; p=0.012), St.Petersburg (MD=4.812; p=0.023), the Udmurtia (MD=7.284; p <0.001) and the Sakha (MD=3.869; p=0.003).

**Conclusions:**

Residents from Russia’s regions with different ethno-cultural composition of population demonstrate some difference in mental well-being measures that may be caused by sociocultural factors. The reported study was funded by the Russian Foundation for Basic Research, project number 17-29-02506.

